# Systemic clinical-grade recombinant decorin reduces disease burden and fibrosis in advanced recessive dystrophic epidermolysis bullosa

**DOI:** 10.1016/j.omta.2026.201737

**Published:** 2026-04-15

**Authors:** Christine Gretzmeier, Bing Hang, Gerhard Sengle, Christopher Phillips, Ben Buer, Sarah Fletcher, Arseniy Belov, Gregory Bleck, Ian J. Collins, Jürgen Brinckmann, Mark P. de Souza, Hal Landy, Alexander Nyström

**Affiliations:** 1 Department of Dermatology, Faculty of Medicine, Medical Center – University of Freiburg, Freiburg, Germany; 2 Faculty of Biology, University of Freiburg, Freiburg, Germany; 3 Department of Pediatrics and Adolescent Medicine, Faculty of Medicine and University Hospital Cologne, University of Cologne, Cologne, Germany; 4 Center for Biochemistry, Medical Faculty, University of Cologne, Cologne, Germany; 5 Center for Molecular Medicine Cologne (CMMC), Medical Faculty, University of Cologne, Cologne, Germany; 6 Cologne Center for Musculoskeletal Biomechanics (CCMB), Cologne, Germany; 7 Cologne Excellence Cluster on Cellular Stress Responses in Ageing-Associated Diseases (CECAD), University of Cologne, Cologne, Germany; 8 Kamal Therapeutics, Hingham, MA, USA; 9 Pace Laboratories, Woburn, MA, USA; 10 Catalent Pharma Solutions Inc. Madison, Madison, WI, USA; 11 Department of Dermatology, University of Lübeck, Lübeck, Germany

**Keywords:** Decorin, dystrophic epidermolysis bullosa, collagen VII, collagen, portein therapy, extracellular matrix, fibrosis, proteoglycan

## Abstract

Recessive dystrophic epidermolysis bullosa (RDEB) is a skin and epithelial fragility disease that progresses with severe fibrosis. This fibrosis is debilitating and promotes the development of aggressive squamous cell carcinomas (SCCs). Effective management of fibrosis may improve quality of life in RDEB and reduce the risk of high-risk SCCs. Decorin, a small leucine-rich proteoglycan, has natural fibrosis-limiting activity. Higher expression of decorin has been linked to milder RDEB, and decorin has shown anti-fibrotic effects in multiple models of experimentally induced fibrosis. However, a short circulating half-life, manufacturing challenges, and broad target engagement have limited the development of systemic decorin therapy. Here, we describe scalable production of recombinant human decorin protein core (rh Decorin) and test repeated intravenous administration in a murine model of advanced RDEB. We show that rh Decorin accumulates in the skin and other injured sites and improves RDEB-related signs. Systemic administration enables decorin-mediated direct and indirect inhibition of TGF-β activity in peripheral tissues. Characterization of the dermal collagen matrix revealed evidence of remodeling capacity of fibrotic lesions in RDEB when cellular contractile activities are targeted, providing an explanation for the anti-fibrotic effects of rh Decorin in advanced disease. These results support further evaluation of intravenous rh Decorin for the treatment of RDEB, especially for fibrotic manifestations.

## Graphical abstract

**Figure:**
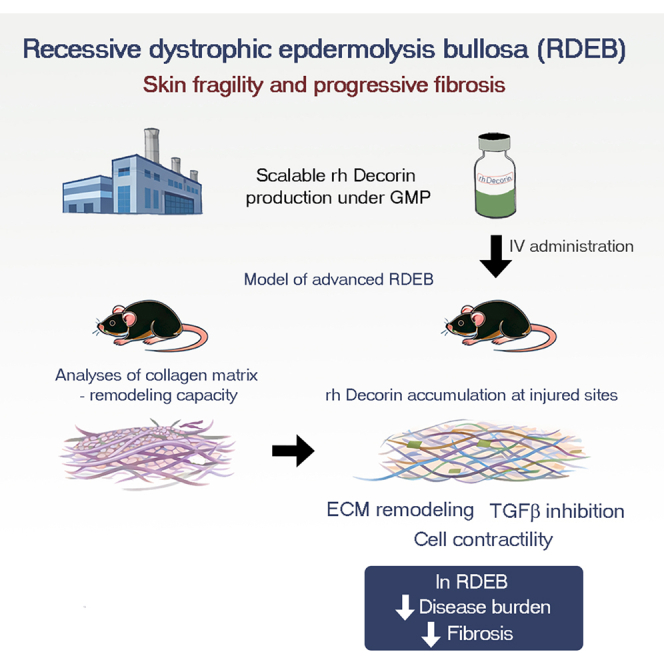

## Teaser

Gretzmeier and colleagues establish scalable production of GMP-grade recombinant human decorin (rh Decorin) and show that systemic delivery limits fibrosis in a model of advanced recessive dystrophic epidermolysis bullosa (RDEB). By accumulating in injured tissue, rh Decorin evokes matrix remodeling and inhibits cellular contractility, supporting its therapeutic potential in RDEB.

## Introduction

Recessive dystrophic epidermolysis bullosa (RDEB) is a severe genetic skin blistering disease associated with a high wound burden, heavy, disabling fibrosis, and the occurrence of high-risk cutaneous squamous cell carcinomas (cSCC). It is caused by biallelic pathogenic variants in *COL7A1*, which encodes the anchoring fibril-forming collagen, collagen VII.^1^ Anchoring fibrils are essential for firm adhesion of the epidermis to the underlying dermis and, consequently, loss of functional anchoring fibrils, which occurs in RDEB, results in skin that blisters upon minor frictional encounters. Such challenges unleash a pathogenic fibro-inflammatory cascade.^2^ Targeting this cascade with agents that limit fibroblast and inflammatory activities, such as losartan and mesenchymal stromal cells, has provided clinical support for the effectiveness of symptom-relief therapies for the management of RDEB.^3^^,^^4^

There has been significant progress in developing localized wound-healing therapies for RDEB with three therapies—two gene therapy approaches and a wound symptom-relief treatment—receiving FDA approval during the past 3 years.^5^^,^^6^^,^^7^ While the approved therapies could potentially also reduce scarring and fibrosis,^8^ there remains a high unmet need for the management of systemic manifestations of RDEB. Fibrosis in RDEB is not limited to the skin, but progressive fibrosis also occurs at other sites, including the eye, oral, esophageal, and anal mucosa. Furthermore, evidence points to the fact that the consequences of chronic tissue damage, inflammation, and fibrosis underlie the conversion of cSCC to high-risk cSCC in RDEB.^9^ Consequently, for RDEB, there is an urgent need to provide efficacious systemic management of fibrosis. Some approaches, such as losartan, have shown promising results in clinical trials, suggesting that delay or even some reversal of fibrosis may be possible in RDEB.^4^ However, heterogeneity in response between individuals has been observed, and thus additional approaches are needed, potentially allowing for combinatory treatment regimens adapted to disease severity and stage.

Through analyses of differentially affected monozygotic twins with RDEB, followed by studies in preclinical models, the extracellular matrix (ECM) protein decorin appeared as a candidate for fibrosis management in RDEB.^10^^,^^11^ Decorin belongs to a class of proteins—the small leucine-rich proteoglycans^12^—with ECM and direct and indirect cell-instructive activity. Deficiency of decorin in humans and mice presents with altered organization of the fibrillar collagen matrix, providing evidence that a major physiological activity of decorin is to support the organization of fibrillar collagens.^13^^,^^14^ However, decorin, apart from fibrillar collagens, has been reported to interact with a large number of proteins, including growth factor receptors and growth factors.^15^ One prominent activity of decorin is the downmodulation of cellular responses to chronic injury, such as during neoplasia or fibroproliferative diseases.^10^^,^^16^^,^^17^ Decorin replacement, via viral expression or recombinant protein infusions, has shown therapeutic efficacy in preclinical models of various solid tumors and fibrosing diseases. The modes of action assigned to decorin in mediating such effects are multifaceted, including interference with growth factor receptor signaling, promoting cell-detrimental macroautophagy, leading to cell death, and angiogenesis inhibition.^18^^,^^19^ In the context of fibrosing diseases, a major focus has been on the ability of decorin to sequester and limit transforming growth factor β (TGF-β), as identified by the Ruoslahti lab.^20^ Studies of response to kidney injury in decorin-deficient mice support decorin as a natural repressor of TGF-β activity.^21^ However, these and subsequent studies also point to the contextual effects of decorin in fibrosis and wider mechanisms beyond simply direct decorin-TGF-β interactions involved in decorin-mediated repression of TGF-β activity.^21^^,^^22^ Namely, decorin might protect collagen fibrils from proteolysis^21^ and support the organization of fibrillin microfibrils,^22^ which are potent regulators of TGF-β bioavailability.^23^

Seminal work from Odorisio and colleagues on a pair of monozygotic twins with disparate severity of RDEB identified decorin as a potential natural modifier of RDEB.^11^ The milder affected twin had higher levels of decorin production in dermal fibroblasts, which was linked to a reduction of pro-fibrotic traits *in vitro*. Subsequent studies assessed the potential of decorin for disease management of RDEB using a mouse model of severe generalized RDEB—the collagen VII hypomorphic mouse.^10^ Systemic injections of lentivirus expressing human decorin in RDEB mouse pups significantly increased pup survival and reduced the formation of fibrosis-driven mutilating or mitten deformities of the forepaws.^10^ In follow-up work by the Järvinen and Liao laboratories, systemic injections of recombinant human decorin via intraperitoneal administration every other day after birth extended the survival of conventional *Col7a1*-knockout mice compared to a group receiving PBS alone.^24^ However, a specific disease-ameliorating effect of decorin injections could not be disclosed, as historical data from injections with human serum albumin indicated a comparable effect on the survival of *Col7a1*-knockout mouse pups with both treatments and decorin injections.^24^ By attaching a wound-seeking peptide, tCRK, to decorin, the effect on survival was enhanced, and this enhancement was linked to improved deposition of the decorin chimera (decorin-tCRK) in the skin.^24^ These studies suggested that systemic recombinant decorin infusion could be effective for the management of RDEB if sufficient decorin is provided to the tissue.

Decorin naturally exists in two forms: a proteoglycan carrying one chondroitin or dermatan sulfate glycosaminoglycan (GAG) chain or as a non-GAGylated protein core.^25^ The GAG chain of decorin appears to support collagen fibrillogenesis, whereas most growth factor-binding and receptor-binding activities, including cell proliferation-inhibiting TGF-β sequestering, occur via the protein core.^15^^,^^20^^,^^26^ Because GAG chains are bulky, relatively rigid, and charged, we reasoned that they could limit the tissue distribution of decorin. In addition, GAG chains could pose a challenge from a manufacturing and regulatory perspective due to their inevitable heterogeneity in composition.^27^ Expression of decorin in mammalian cells at the levels required for scalable recombinant protein production leads to highly elevated secretion of non-GAGylated decorin, further increasing species heterogeneity.^28^^,^^29^ To circumvent such challenges, we used a pharmaceutical-grade human recombinant decorin, in which its N-terminal serine (34), which is the GAG chain attachment site, had been mutated. An additional advantage of the removal of the GAG chain from decorin was that expression of the decorin protein core (without the GAG chain) was possible without any effect on cell proliferation.^30^ This enabled the scale-up and manufacture of pharmaceutical-grade decorin^30^ to provide clinical trial supply in compliance with good manufacturing practices (GMP).

In previous work on intravenously injected collagen VII protein-replacement therapy for RDEB, we had observed that collagen VII accumulated in injured tissue^31^; similar observations have been made for systemically administered decorin in other disease models.^32^ Based on this, we reasoned that repeated systemic administration of recombinant human decorin protein core (rh Decorin) would lead to accumulation of therapeutically active amounts of decorin in chronically injured tissues. Toward this end, we assessed long-term, repeated systemic rh Decorin administration in adult collagen VII hypomorphic, RDEB-model mice, which allowed for monitoring of signs associated with the progression of RDEB.^33^ Despite rapid clearance from circulation, rh Decorin effectively alleviated disease in RDEB-model mice, prompting weight gain and delaying the development of fibrotic mitten deformities. Mechanistically, the effect on fibrosis appeared to occur via accumulation of rh Decorin in chronically injured tissue, leading to downmodulation of pro-fibrotic activities, including the TGF-β pathway. Our studies provide support for systemic rh Decorin for the management of RDEB and other fibrosing diseases progressing through similar contexts.

## Results

### Production in CHO cells of human recombinant decorin protein core and characterization

Decorin has been shown to be effective in many preclinical models of fibroproliferative disease.^16^

Despite this, decorin-based therapies have not reached clinical use. One major hurdle toward the development of therapies based on recombinant decorin has been the scalability and quality assurance of manufacturing. A specific concern has been the GAG chain, which inherently causes heterogeneity of recombinant decorin when expressed in cell lines. Since most potential fibrosis-limiting activity of decorin is attributed to the protein core—as demonstrated by interaction studies and confirmed by *in vivo* activity of non-GAGylated decorin^15^^,^^32^—we chose to express recombinant non-GAGylated decorin to simplify production and reduce potential regulatory concerns. Furthermore, the aim of decorin-based fibrosis-limiting therapies is not to replace endogenous decorin but to therapeutically harness the anti-fibrotic activity of the decorin core. Re-analysis of previous mass spectrometry (MS)-based proteomic data from adult (10-week-old) murine whole back skin^33^ revealed that collagen VII deficiency was not associated with reduced decorin levels (Figure S1). Thus, the potentially lower ability of non-GAGylated decorin to regulate collagen fibril organization, compared with its GAGylated counterpart,^26^ is unlikely to represent a major concern. Moreover, previous studies using recombinant non-GAGylated decorin have demonstrated that fibrillar collagen-organizing capacity is retained.^34^ Indeed, the reduced molecular bulk may even enhance tissue diffusion and thereby improve therapeutic efficacy. To facilitate the production of non-GAGylated decorin, the GAG attachment site in human decorin—serine 34 (serine 4 in the mature protein)—was substituted with alanine. To enable upscaling and support a straightforward regulatory and commercial pathway, Chinese hamster ovary (CHO) cells were used for the production of recombinant human decorin^S34A^.

**Figure 1:**
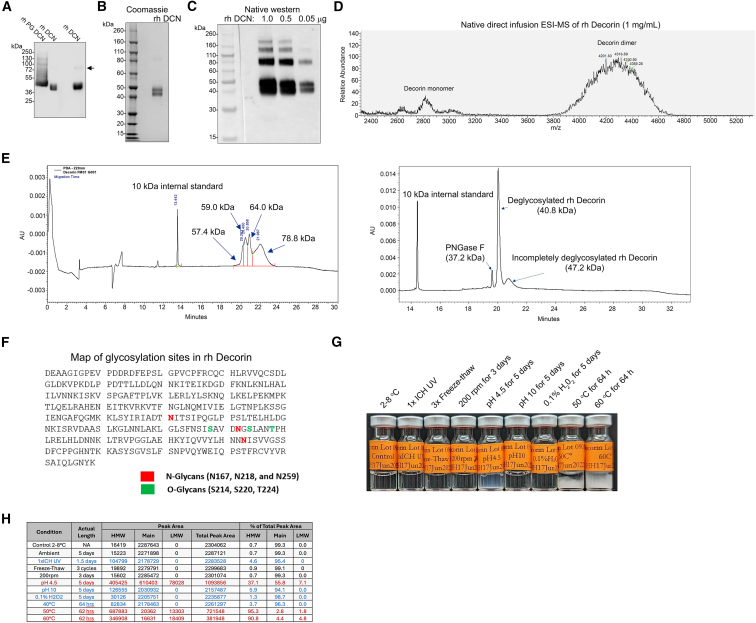
Characterization of CHO-produced decorin protein core (A) Western blot of *in vitro* expressed wild-type proteoglycan (PG) decorin and rh Decorin Arrow indicate dimer formation in rh Decorin. (B) Commassie-stained gel of rh Decorin run under reducing conditions; note the appearance of a double band. (C) Western blot of rh Decorin probed with an anti-decorin antibody; the blot indicates the presence of decorin dimers and multimeric forms. (D) Spectra of ESI-MS analysis of direct infusion of native rh Decorin, revealing the presence of decorin dimers and a broad peak corresponding to both monomeric and dimeric rh Decorin. (E) Capillary electrophoresis analysis of denatured rh Deocrin before (left-hand side) and after deglycosylation (right-hand side), revealing a shift to a sharp peak of 40.8 kDa after deglycosylation. (F) Map of glycosylation sites detected by mass spectrometry analyses of rh Decorin. (G) and (H) Stability assay of rh Decorin subjected to the indicated challenges. (G) shows macroscopically visible precipitation of rh Decorin, indicative of denaturation. (H) summarizes characteristics obtained by size-exclusion chromatography; black text shows conditions determined to be stable, blue text shows conditions leading to partial denaturation, and red shows conditions leading to significant denaturation of rh Decorin.

Western blotting under reducing conditions of mammalian cell-derived, *in vitro*-expressed wild-type decorin and recombinant human decorin S34A (hereafter referred to as rh Decorin) revealed that wild-type decorin, as expected for *in vitro*-expressed GAGylated decorin, partially migrated as a smear^24^^,^^28^ (Figure 1A). In contrast, rh Decorin migrated as a broad double band with no higher-molecular-weight smear; at higher concentrations, a band suggestive of dimer formation was visible (Figure 1A). Coomassie blue staining of SDS-PAGE gels run under reducing conditions showed that rh Decorin migrated as a double band between 40 and 50 kDa. The strong Coomassie blue staining of rh Decorin further supported the absence of GAG chain substitution^35^ (Figure 1B). Native western blotting revealed that, under non-reducing conditions, rh Decorin migrated with additional distinct bands of higher molecular weight, suggesting dimerization and additional multimerization (Figure 1C). This is in accordance with the high propensity of decorin to form dimers,^36^ which are nevertheless relatively weakly associated and reversable, but may be unable to sustain interactions with interaction partners, including fibrillar collagens.^37^ A high ratio of dimer formation in 1 mg/mL concentrated fractions of rh Decorin was confirmed by native electrospray ionization MS (ESI-MS) (Figure 1D).

The two distinct bands of decorin on reducing gels suggested differential post-translational modifications, of which altered glycosylation appeared most likely.^28^ Toward this end, for analytical purposes, rh Decorin was treated with PNGase F to remove N-linked glycans. To increase efficacy, rh Decorin was denatured prior to incubation PNGase F. Subsequent capillary electrophoresis analysis of reduced rh Decorin revealed that deglycosylation evoked a shift of rh Decorin from a broad multipeak spanning calculated weights of 57–79 kDa to a sharp single peak of 40.8 kDa (Figure 1E). To gain further insights into the glycosylation of recombinant human decorin, we performed label-free liquid chromatography-MS (LC-MS) with data-dependent acquisition and higher-energy collisional dissociation fragmentation to identify and quantify glycans. These analyses revealed N-glycosylation of three of the four previously reported glycosylated asparagines^37^ (Figure 1F). In addition, three putative O-glycosylation sites were identified (Figure 1F). Importantly, analyses of two different batches of CHO-expressed rh Decorin pointed to relatively low batch variation (Figure S2; Tables S1 and S2).

The protein core of decorin can bind directly to collagen I, although with reduced affinity compared to GAGylated decorin.^38^ For an initial assessment of functionality, we evaluated the binding of rh Decorin to immobilized, acetic acid-dissolved collagen I from calf skin using a solid-phase assay. In this system, rh Decorin showed a half-maximal interaction at 26.6 nM (Figure S3A), which is within the range of the previously reported affinity of the decorin protein core for collagen I^38^. A well-established function of decorin is the regulation of collagen fibrillogenesis; however, previous studies have shown that the presence of a GAG chain strongly supports this activity.^26^ We nevertheless tested the ability of rh Decorin to inhibit collagen fibrillogenesis *in vitro*. In contrast to a previous study, which reported no inhibitory effect of the decorin protein core at the concentration used,^26^ we observed that rh Decorin showed some delaying effect on collagen fibrillogenesis (Figure S3B). Thus, rh Decorin retains well-characterized biological functions of decorin.

Next, we assessed the stability of rh Decorin in a forced degradation study by exposing it to various challenges, followed by analyses by size exclusion chromatography. The following conditions were tested: rh Decorin stored at 2°C–8°C, stored at room temperature for 5 days, at 40°C for 64 h, at 50°C for 62 h, and at 60°C for 62 h; 1× UV exposure in accordance with International Council for Harmonisation (ICH) guidelines for UV testing for 1.5 days; 3 freeze-and-thaw cycles; agitation at 200 rpm 3 days; storage at pH 4.5 for 5 days; at pH 10 for 5 days; or in 0.1% H_2_O_2_ for 5 days. The analyses revealed that rh Decorin stored at 2°C–8°C or at room temperature, exposed to agitation or multiple freeze-and-thaw cycles, remained stable, as determined by no visible aggregation, a highest percentage of the main peak, and no loss of concentration (Figures 1G and 1H). Exposure to temperatures above ambient and non-physiological pH resulted in varying degrees of aggregate formation (Figures 1G and 1H).

Collectively, we established reproducible large-scale production of rh Decorin in CHO cells, and this, together with the stability rh Decorin during long-term storage at 2°C–8°C or even at room temperature, enables a reliable clinical trial supply of rh Decorin.

### Repeated systemic administration of recombinant human decorin protein core improves indicators of overall health status in RDEB-model mice

Previous studies had indicated that systemic supplementation of decorin could improve health and reduce fibrosis progression in collagen VII-deficient mice.^10^^,^^24^ Although direct administration of decorin as a protein has been effective in limiting fibrosis in various experimental models, its short systemic half-life has been viewed as a challenge to achieve effective systemic fibrosis-modulating therapies based on recombinant decorin.^39^ Furthermore, the efficacy of recombinant decorin infusions in a context of naturally developing fibrosis has not been investigated. We reasoned that the short systemic half-life of intravenously administered decorin,^40^^,^^41^ combined with its ability to accumulate in damaged tissue,^24^^,^^32^ could instead be an advantage, potentially increasing safety and decreasing off-target effects.

**Figure 2:**
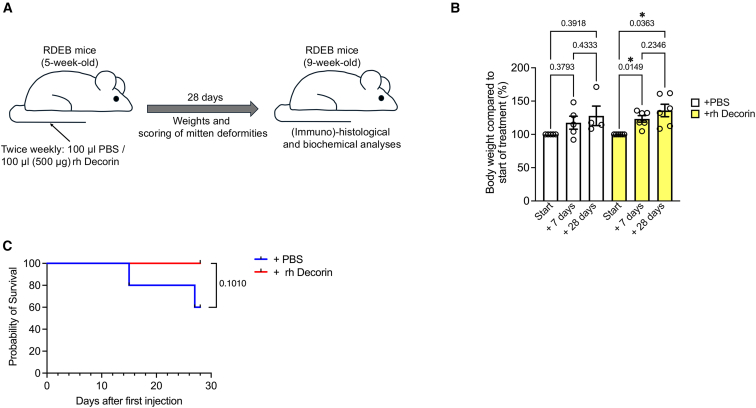
Intravenous injections of rh Decorin improves the overall health status of RDEB-model mice (A) Schematic illustration of the treatment regimen of RDEB-model mice with either PBS or rh Decorin. (B) Body weights of PBS- or rh Decorin-treated RDEB mice, expressed as the percentage of the weight at the start of treatment. (C) Kaplan-Meier curve of survival during the treatment and observation period. Statistical analyses: 2B, ANOVA mixed-effects analysis; 2C, log-rank (Mantel-Cox) test. *p* values as indicated. ∗*p* < 0.05.

All of the above led us to evaluate the efficacy of repeated rh Decorin infusions as a disease-modulating therapy in RDEB-model mice. To position the studies in a clinically relevant setting modeling treatment of a target population of children and young adults, we started treatment when the RDEB-model mice were 5 weeks old (Figure 2A). At this age, RDEB mice generally display somewhat advanced disease, with the first macroscopic signs of the formation of mitten deformities, which in children with RDEB tend to occur from a few years of age. For the studies, a control group of RDEB-model mice received PBS injections (Figure 2A), whereas the rh Decorin-treated group received 500 μg rh Decorin in PBS. This dose was selected as it was the highest feasible dose, based on the solubility of rh Decorin and the maximal injectable volume in RDEB-model mice. The mice received twice-weekly injections for 28 days. Repeated rh Decorin injections were well tolerated in RDEB-model mice, which displayed no macroscopic signs of adverse reactions to the treatment. In contrast, they significantly gained weight during the treatment, whereas this effect was not observed with PBS injections alone (Figure 2B). Furthermore, while all rh Decorin-treated mice survived until the planned end of the experiments, two PBS-treated mice deteriorated in health, met termination criteria, and had to be euthanized during the observation period (Figure 2C). Collectively, these observations indicate that repeated systemic injections of rh Decorin in RDEB-model mice do not cause any major adverse events and appear to improve the overall health status of RDEB-model mice.

### Systemically administered recombinant human decorin protein core deposits in injured tissue

**Figure 3:**
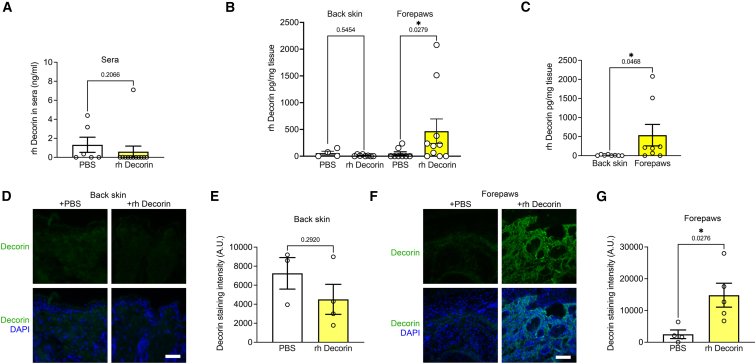
Intravenously injected rh Decorin deposits in injured RDEB mouse skin (A–C) ELISA measurement of human decorin levels with an assay specific for human in (A), sera; B, back skin; and C, forepaw skin from PBS- and rh Decorin-treated RDEB mice. The mice were injected with 500 μg rh Decorin 30 min prior to collection of sera. Values represent two technical duplicates per mouse. (D-G) staining of back skin and forepaws from RDEB mice treated for 4 weeks with either PBS or rh Decorin, using an antibody that preferentially detects human decorin. (D) back skin; (F) forepaws. (E) and (G) quantification of fluorescence staining intensity corresponding to samples in (D) and (F), respectively. Four to six mice per group were analyzed, with four fields quantified per mouse. Data are presented as mean ± SEM, with values for individual fields shown. Nuclei were visualized with DAPI (blue). Scale bars, 100 μm. Statistical analysis: (A) Mann-Whitney test; (B) Kruskal-Wallis test; (C) Mann-Whitney test; and (E) and (G) Student’s unpaired *t* test. *p* values as indicated. ∗*p* < 0.05.

Next, to understand whether rh Decorin exerted its effect at the tissue or systemic level, we first analyzed the distribution of human decorin in the body after systemic injection of rh Decorin. We were unable to detect human decorin in the sera from RDEB mice 30 min after infusions with 500 μg rh Decorin (Figure 3A). This is in accordance with previous reports that systemically injected decorin was rapidly cleared from the circulation.^40^^,^^41^ This suggested that decorin exerted its effects in RDEB at the tissue level rather than at the circulatory level. Therefore, we analyzed skin samples from different body sites for human decorin by ELISA. We used back skin, which is protected from damage and displays slow disease progression in RDEB-model mice^33^ and skin from forepaws, which, in contrast, displays rapid injury-driven disease, as manifested by heavy fibrosis.^33^ In back skin from mice treated with repeated rh Decorin injections for 28 days, we could not observe a detectable accumulation of human decorin (Figure 3B). However, in tissue lysates from the forepaws of rh Decorin-treated RDEB mice, we detected human decorin accumulation (Figure 3B). Head-to-head analyses of human decorin deposition in forepaw skin versus back skin also revealed significantly more human decorin content in forepaws compared to back skin (Figure 3B). Although, on average, significantly increased, there was, nevertheless, a relatively high spread in the measured decorin levels in forepaws from different rh Decorin-injected mice, indicating variability in uptake among individual mice. Western blotting of forepaw lysates confirmed these observations (Figure S4A) and, importantly, showed that rh Decorin accumulation also occurred in the esophagus and tongue—two other organs frequently damaged in RDEB (Figures S4B and S4C). Again, there was a notable difference in tissue uptake of decorin among individual mice.

The observation of human decorin deposition in sites of injured tissue was encouraging. However, we reasoned that integration of injected rh Decorin in the dermal ECM could limit decorin solubility and detectability in non-reducing tissue lysates, as used for the ELISA measurements. Therefore, we analyzed decorin deposition by staining the tissues with an antibody with higher affinity for human decorin compared to murine decorin. Staining and quantification of staining intensity confirmed significant deposition in forepaws, whereas human decorin could not be detected in back skin (Figures 3D–3G). Human decorin in forepaws was deposited in the dermis and was associated with the dermal ECM (Figure 3F). Thus, we concluded that systemically administered rh Decorin deposits at injured sites, which are also the sites of actively ongoing fibrosis.

### Systemically administered recombinant human decorin protein core limits fibrosis

**Figure 4:**
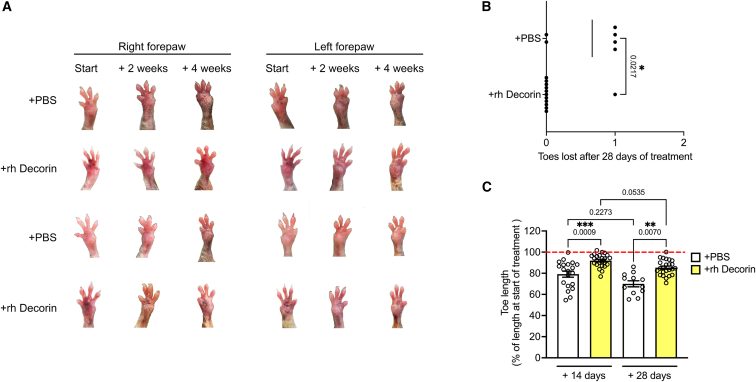
Intravenously injected rh Decorin slows formation of mitten deformities in RDEB-model mice (A) Photograph of forepaws, right and left as indicated, from representative mice at treatment start and after 2 and 4 weeks of treatment. (B) Quantification of the number of lost toes on forepaws during 28 days of treatment. (C) Measurement of the lost toe length of the two most prominent toes on mouse forepaws as previously described.^16^ Statistical analyses: (B) Mann-Whitney test; (C) Kruskal-Wallis test. *p* values as indicated. ∗*p* < 0.05; ∗∗*p* < 0.01, ∗∗∗*p* < 0.001.

**Figure 5:**
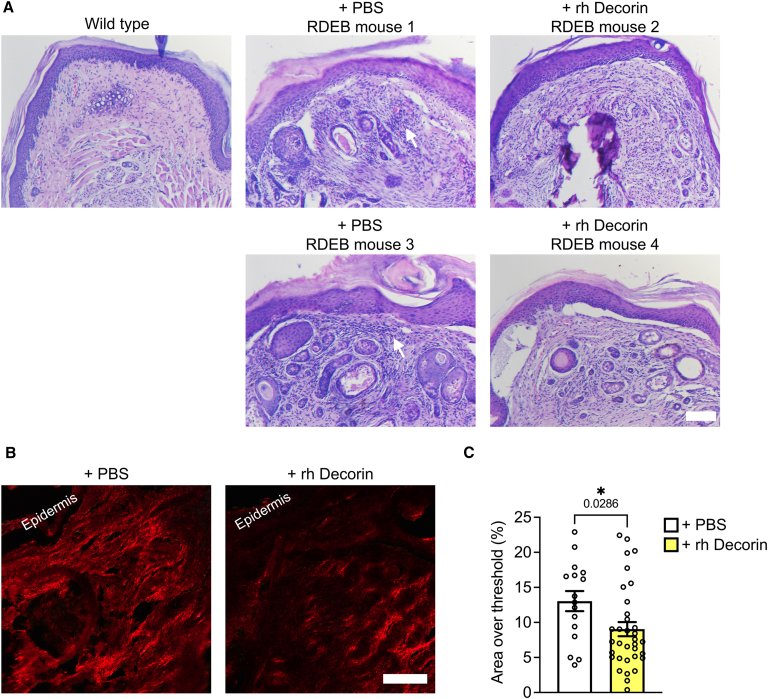
Intravenously injected rh Decorin improves the appearance of forepaw dermis and affects fibrillar collagen alignment (A) Hematoxylin and eosin staining of forepaws, as indicated, from mice that had received twice-weekly injections of PBS or rh Decorin for 4 weeks (28 days). Arrows indicate increased cellular density in dermis. Scale bars, 100 μm. (B) Picrosirius red staining of forepaws as in (A), visualized under cross-polarizing light. (C) Quantification of red staining intensity over a digitally applied threshold. Four to six mice per group were analyzed, with four fields quantified per mouse. Data are presented as mean ± SEM, with values for individual fields shown (Figure 4C). Student’s unpaired *t* test. *p* values as indicated. ∗*p* < 0.05.

Having established that systemically supplied rh Decorin accumulates at injured sites, we subsequently analyzed the outcome of fibrosis at such locations, with a focus on forepaws. Formation of mitten deformities of forepaws in RDEB mice is driven by tissue contraction and fibrosis.^33^^,^^42^ Macroscopic analyses of the progression of mitten deformities revealed that rh Decorin treatment stalled the progression of macroscopic fibrosis, leading to significantly fewer toes lost and less average toe length shortening (Figure 4). Notably, the effect of rh Decorin was rapid, with a significant effect on preservation of toe length being observed after only 2 weeks (Figure 4C). On a histological level, forepaws from rh Decorin-treated RDEB mice after 28 days of treatment displayed signs of reduced dermal cellularity compared to forepaws from PBS-treated mice (Figure 5A). Moreover, the general organization of the dermis appeared improved in rh Decorin-treated mice; however, the treatment did not strengthen epidermal-to-dermal cohesion (Figure 5A). Notably, analyses of the dermal collagen matrix organization via picrosirius red staining visualized under polarizing light,^33^ revealed, by showing lower intensity in the red channel, that rh Decorin treatment reduced the parallelism of collagen fibrils or their thickness (Figures 5B and 5C). Taken together, repeated systemic infusions of rh Decorin reduced the progression of macroscopic and histologically detectable fibrosis in RDEB-model mice.

### Decorin anti-fibrotic effect involves TGF-β signaling and tissue contractility reduction, enhanced by RDEB dermal ECM properties

The rapid anti-fibrotic response to rh Decorin indicated that the effect was mediated through increasing tissue relaxation via reduction of fibroblast activity and targeting of provisional ECM organizers, rather than by remodeling of the collagen matrix.^43^ For relief of cellular contraction to result in such effects, this would imply a deformable interstitial ECM. Toward this end, we measured the general protein composition and collagen matrix crosslinks of skin from wild-type and age-matched adult RDEB-model mice. Due to limitations in the amount of skin that could be obtained from the forepaws—restricting reliability and reproducibility—we analyzed back skin from 9- to 11-weeks-old wild-type and RDEB mice, an age at which the back skin of RDEB mice also shows molecular signs of fibrosis.^33^ Wild-type and RDEB mouse skin did not differ in the total amounts of non-collagenous and collagenous proteins (Figure S5A). However, RDEB mouse skin was significantly less hydrated (Figure S5B), leading to a significantly higher collagen abundance per wet tissue weight (Figure S5C). This could result in increased collagen fibril density and contribute to the increased picrosirius red staining signal under polarizing light generally observed in RDEB.^4^^,^^44^^,^^45^ Intriguingly, hydroxylysylpyridinoline (HP) and dihydroxylysinonorleucine (DHLNL) crosslinks of collagens were significantly lower in RDEB mouse skin (Figure S5D), and there was a significant decrease in the DHLNL-to-immature HLNL crosslink ratio (Figure S5E), which is increased in other fibrotic conditions.^46^ Collectively, this suggests a reversibility of the collagen matrix in RDEB.^47^

**Figure 6:**
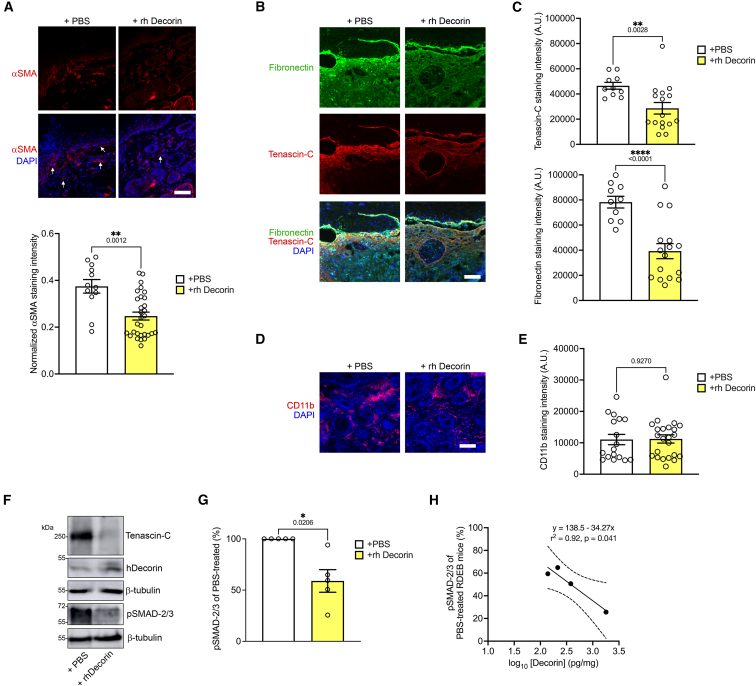
Intravenously injected rh Decorin reduces deposition of fibronectin and tenascin-C and canonical TGF-β activity but does not affect inflammatory cell abundance in RDEB mouse forepaws (A) Staining of forepaws from RDEB-model mice treated with twice-weekly injections of PBS or rh Decorin for 4 weeks (28 days): αSMA (red) (top) and staining intensity of papillary dermal cells quantified and normalized to DAPI staining (bottom). Arrows indicate αSMA-positive cells in the papillary dermis. (B) Staining for fibronectin and tenascin-C, which are increased during RDEB fibrosis. (C) Quantification of fluorescence staining intensity of stains as in (B). (D) Staining of forepaws from RDEB mice treated for 4 weeks with either vehicle or rh decorin, using a granulocyte marker Cd11 b. (E) Quantification of staining as described in (D). (F) Western blot of protein lysates from forepaws from mice as in (A). The blots were probed with antibodies against tenascin-C, phospho(p)SMAD-2/3, human decorin (hDecorin), and β-tubulin to indicate loading. The lanes for hDecorin and one β-tubulin blot are also shown in Figure S4A. (G) Quantification of blots as in (F), rh Decorin-treated mice were compared to PBS-treated mice, for which *p*-SMAD-2/3/β-tubulin was set to 100%. The graph shows values per each such comparison per blot. (H) *p*-SMAD-2/3 abundance plotted against human decorin content measured by ELISA as in 3C; dotted lines indicate 95% confidence intervals. For (A–E), four to six mice per group were analyzed, with four fields quantified per mouse. Data are presented as mean ± SEM, with values for individual fields shown. Statistical analysis was performed using Student’s *t* test (unpaired for (A), (C), and (E) and paired (G) and simple linear regression (H)*. p*-values are indicated. Nuclei were visualized with DAPI (blue). Scale bars, 50 μm (A, D) 100 μm (B). Data are presented as mean ± SEM. *p* values as indicated. ∗*p* < 0.05, ∗∗*p* < 0.01,∗∗∗∗*p* < 0.0001.

Following this, we examined differences in soft-tissue contractile ability in PBS-treated vs. rh Deocrin-treated paws using alpha smooth muscle actin (αSMA) as a biomarker and proxy of contractile activity. Since αSMA is expressed by smooth muscle cells around vessels, general analyses of αSMA abundance by western blotting are of limited informative value. Therefore, we focused on regional analyses using immunostaining of the upper papillary dermis in toes, which are sites that are exposed to constant frictional challenges and consequently become chronically damaged and are generally the first sites affected by scarring and fibrosis.^44^ In forepaws from PBS-treated mice, there was an abundance of αSMA-positive cells at the papillary dermis toward the fragile epidermal-dermal interface (Figure 6A). Notably, in forepaws from rh Decorin-treated mice, the αSMA cell positivity was significantly diminished (Figure 6A). This indicates a more relaxed tissue after rh Decorin treatment, contributing to the observed altered appearance of the collagen matrix (Figure 5B).

We continued to investigate changes in the expression of proteins that are key ECM organizers known to be active in fibrosis. We focused on fibronectin and tenascin-C, as they have previously been shown in multiple studies to be altered in fibrotic RDEB skin, and their alterations have been associated with fibrosis-limiting responses.^33^^,^^44^^,^^48^ Staining of forepaw sections for both proteins showed that samples from rh Decorin-treated RDEB mice, compared to PBS-treated RDEB mice, displayed a significant reduction in the staining intensity of fibronectin and tenascin-C (Figures 6B and 6C). These observations, together with the reduction in αSMA, align with the relaxation of the collagen matrix in rh Decorin-treated mice and provide further support for the fibrosis-delaying effect of systemic rh Decorin treatment for RDEB.

Studies from others and us have provided support that fibrosis in RDEB is driven through pro-inflammatory, immunity-activating fibroblasts, which subsequently disseminate fibrosis via TGF-β-dependent and independent mechanisms.^33^^,^^48^^,^^49^^,^^50^ Decorin is generally considered to be pro-inflammatory through its core acting as an endogenous TlR2 and TLR4 ligand;^51^ nevertheless, its effect on inflammation is contextual, and it may, via signaling through other receptors, suppress pro-inflammatory gene expression.^52^ There was no noticeable difference in CD11b-positive leukocytes, macrophages, and neutrophils in forepaw skin from PBS- and rh Decorin-treated RDEB mice (Figures 6D and 6E; Figure S6), indicating no drastic influences of the pro-inflammatory activity of rh Decorin. This was in accordance with previous observations from viral expression of human decorin in RDEB-model mice.^10^ Consequently, we then turned to assessing TGF-β signaling as a second important target of decorin.^15^^,^^27^ In contrast to the non-noticeable effect on inflammation, we detected a potent and significant repression of canonical TGF-β signaling activity (phospho-SMAD-2^Ser465/467^/3^Ser423/425^) in forepaws from rh Decorin-treated RDEB-model mice (Figures 6F and 6G). Importantly, this correlated with a reduction in markers of fibrosis remodeling, and there was a correlation between decorin abundance and lowering of TGF-β activity, as assessed by pSMAD-2/3 (Figure 6H). Decorin can limit TGF-β signaling by direct interaction with TGF-βs;^20^ however, observations suggest that it can also indirectly affect TGF-β activity by altering TGF-β bioavailablity through regulation of fibrillin-1.^22^^,^^53^ Furthermore, in previous studies on RDEB, we observed morphological alterations in fibrillin fibers both in the skin and in those deposited by cultured fibroblasts. Therefore, we analyzed the abundance and organization of the fibrillin-1 fiber network in forepaw skin from RDEB-model mice treated with either rh Decorin or PBS. In the forepaws of rh Decorin-treated RDEB mice, there was a slight but significant increase in fibrillin-1 abundance compared to PBS-treated controls (Figures S7A and S7B).

Immunofluorescence staining of forepaw sections confirmed previous observations,^48^ showing predominantly condensed fibrillin-1 staining at the dermal-epidermal junction in RDEB-model mice, whereas in wild-type mice, fibrillin-1 fibers were distributed throughout the dermis (Figure S5C). Notably, rh Decorin-treated RDEB mice, while still exhibiting condensed fibrillin-1 staining at the dermal-epidermal junction, displayed improved fibrillin network organization, with distinct fibers extending deeper and more broadly within the dermis (Figure S7C). Quantification of the fibrillin-1-stained area in the forepaw dermis supported these observations (Figure S7D). To support these findings, we analyzed fibrillin-1 synthesized and deposited by RDEB-donor dermal fibroblasts in the presence of absence of rh Decorin. As observed for mice, rh Decorin slightly increased total fibrilin-1 abundance and deposition of fibrillin-1 fibers (Figures S7E and S7F). Since decorin has been described to increase fibrillin-1 through insulin growth factor receptor (IGFR)-stimulated gene expression of *FBN1*,^22^ we analyzed *FBN1* expression. However, in our experiment, rh Decorin did not promote *FBN1* expression (Figure S7G), suggesting that rh Decorin may regulate fibrillin-1 through stabilization rather than gene expression. To further elucidate the fibrosis-limiting mechanisms of rhDecorin, we assessed its effect on baseline TGF-β activity in RDEB-donor fibroblasts at different treatment time points. Interestingly, when fibroblasts were treated the day after seeding—under conditions of low ECM deposition^54^— rhDecorin had minimal effect on pSMAD-2/3 after 1 h of treatment (Figures S7H and S7I). In contrast, daily treatment with rhDecorin for 3 consecutive days, followed by analysis of pSMAD-2/3 on day 4 (24 h after the last treatment), showed potent suppression of pSMAD-2/3 (Figures S7J and S7K). Moreover, in this setting, downstream TGF-β targets, fibronectin and αSMA, were also significantly reduced in rhDecorin-treated cultures (Figures S7J and S7L–S7N). Importantly, this corresponded to a functional reduction in pro-fibrotic, tissue-contractile activity, as assessed by collagen-gel contraction assays (Figures S7O and S7P). These results indicate that rhDecorin exerts a long-lasting suppressive effect on TGF-β activity, which involves the deposited ECM.

To provide further understanding to the fibrosis-limiting mode of actions of rh Decorin, we assessed the effect of rh Decorin on baseline TGF-β activity in RDEB-donor fibroblasts after different time points of treatment. Interestingly, when RDEB-donor fibroblasts was treated the day after seeding, thus in a condition with low ECM deposition,^54^ rh Decorin had a limited effect on pSMAD-2/3 after 1 h of treatment (Figures S7H and S7I). However, daily treatment with rh Decorin for 3 consecutive days, followed by analysis of pSMAD-2/3 on day 4 after seeding (24 h after the last decorin treatment), revealed that rh Decorin potently suppressed pSMAD-2/3 (Figures S7J and S7K). In addition, in this setting, the TGF-β downstream targets fibronectin and αSMA were also significantly reduced in the rh Decorin-treated cultures (Figures S7J, S7L–S7N). Importantly, this also translated into a functional reduction of pro-fibrotic, tissue-contractile activity, as assessed by collagen-gel contraction assays (Figures S7O and S7P). Thus, our data indicate that the suppressive activity of rh Decorin on TGF-β activity is long-lived and involves changes in the ECM.

Collectively, these data indicate that one major mediator of the symptom-relieving effect of systemic rh Decorin in RDEB is interference with TGF-β activity, potentially as the result of several mechanisms working together, which reduce profibrotic ECM modulators, αSMA positivity of fibroblasts, and subsequent tissue contraction.

## Discussion

Here, we show that repeated intravenous administrations of rh Decorin effectively delay disease progression in RDEB-model mice.

There is an abundance of data, starting from the initial studies by Ruoslahti and colleagues in the early 1990s,^17^ showing the fibrosis-limiting potential of decorin.^39^ Studies spearheaded by the Iozzo laboratory have provided conclusive evidence of the efficacy of systemically delivered decorin protein, albeit in the setting of cancer.^16^ Nevertheless, these preclinical studies have so far not been translated into clinical testing or the development of decorin protein-based therapeutics. Multiple reasons account for the challenges in moving decorin from bench to bedside, including concerns about the low systemic half-life of decorin, challenges in reproducible production, and the incompletely understood and broad mechanisms of decorin.^27^^,^^39^

Our study provides a significant advance in bringing decorin protein-based therapies to clinical testing, future approval, and the market. We developed scalable and reproducible production of rh Decorin. The presence of a GAG chain on decorin naturally creates a diversity of recombinantly produced decorin molecules; there are also additional potential limitations to using a GAGylated protein for protein-replacement therapy, including uncontrolled interactions with growth factors and reduced tissue diffusion through steric hindrance. Toward this end, we expressed only the decorin protein core without a GAG chain by mutating the serine-glycine site needed for GAG chain attachment (called rh Decorin). Expression of rh Decorin in CHO cells allowed for scalable production. The aim of the study was not to perform a head-to-head comparison of the anti-fibrotic activities of non-GAGylated and GAGylated decorin but to characterize and investigate the preclinical efficacy of scalable, GMP-produced decorin—rhDecorin—for the treatment of a murine model of advancing RDEB. By careful molecular analyses, we determined that rh Decorin displayed relatively low variation in post-translational modifications between batches, which should be acceptable to regulatory agencies. These variations were mainly attributed to N-linked glycosylation, which nevertheless showed limited differences between the tested batches. A concern of therapies using recombinant proteins is protein stability. Our analyses of protein stability showed that rh Decorin was stable, i.e., did not become denatured, over a range of conditions, including multiple freez-and-thaw cycles, and remained stable at 2°C–8°C for over 3 years, and even at room temperature for over a year. This significantly facilitates shipment to and subsequently storage of rh Decorin at remote locations.

The rapid clearance of intravenously administered rh Decorin from the circulation has been viewed as another potential concern of therapies based on systemic administration of decorin.^39^ We reasoned that the half-life of decorin, once incorporated into tissue, would be substantially longer. Consequently, the effective clearance of decorin from the system could rather be an advantage, favoring safety. It has also been suggested that modifying decorin to enhance targeting of injured tissue could improve its efficacy.^24^^,^^41^ However, modification of natural proteins comes with disadvantages, such as concerns about increasing immunogenicity. From studies on tumors, it was known that uptake of decorin at injured, tumor-bearing sites is high and that, once deposited, decorin remains present in the tissue microenvironment for an extended time.^32^ This is also in line with what we have observed with systemic collagen VII protein-replacement therapy, where intravenously injected recombinant collagen VII effectively deposited at injured sites in RDEB models.^31^ Our data demonstrate that preferential and substantial accumulation of rh Decorin occurs at injured sites, as indicated by the presence of rh Decorin in tissues naturally injured in RDEB-model mice—forepaws, esophagus, and tongue—whereas uptake a back skin, which is normally not wounded in RDEB-model mice, is low. The observed macroscopic and molecular effect on fibrosis strongly suggests that the tissue concentration of rh Decorin achieved by repeated systemic administration is sufficient to evoke a fibrosis-limiting response at such sites. It is important to note that our data do not exclude the possibility that further modifications to decorin, such as attaching a target-seeking peptide, could improve its anti-fibrotic activities *in vivo*.

The primary aim of our studies was to investigate the therapeutic potential of systemic rh Decorin for RDEB. Therefore, we decided to administer the highest feasible dose, which was 500 μg rh Decorin per infusion. The aim was not to find an optimal dose or treatment regimen. As the wound distribution and injury burden in RDEB-model mice and people with RDEB are quite different, dose-reduction or escalation studies in RDEB mice provide limited information toward such ends.

Another challenge hampering the development of a decorin-based therapeutic has been, and continues to be, the incomplete understanding of its mechanism, which are likely indication dependent. Decorin has a large interactome, directly binding collagens, other ECM proteins, growth factors, cytokines, TLR2 and TLR4, and growth factor receptors, including EGFR, cMET, and VEGFR3.^15^^,^^51^^,^^55^^,^^56^^,^^57^ In the context of cancer, initial engagement with receptor tyrosine kinases and TLRs has been postulated to be the main mechanism mediating the anti-tumor activities.^18^ For fibrotic diseases, the focus has been on the ability of decorin to sequester and neutralize the activities of TGF-βs.^20^ The outcome of targeting TGF-β activity is contextual, especially in a complex disease like RDEB, as regulated TGF-β activity is needed for tissue regeneration and regulated immune responses. Too strong targeting of TGF-β could result in impaired tissue regeneration and enhanced pro-inflammatory activity.^58^ Furthermore, controversy surrounds the ability of decorin to modulate TGF-β or the net outcome of decorin on TGF-β activity *in vivo*.^21^^,^^22^ The benefit of targeting TGF-β in a situation of already established fibrotic lesions is also unclear, since a fibrotic ECM is often heavily crosslinked, making it quite inert. Our analyses revealed no apparent effect of rh Decorin on tissue inflammation in RDEB. This is consistent with our previous studies using lentiviral-mediated systemic expression of decorin in RDEB-model mice.^10^ In contrast, there was a negative correlation between rh Decorin levels in tissue and TGF-β activity, as assessed by pSMAD2/3. Interestingly, our *in vitro* analyses, supported by the restoration of fibrillin-1 observed in tissue, suggested that the influence of rh decorin on TGF-β activity could occur through both direct and indirect regulation of TGF-β bioavailability. The interaction sites of TGF-β in the decorin protein core have been roughly mapped, containing one high-affinity site and one site with lower affinity,^59^ with the higher-affinity site not competing with collagen I binding. Thus, collagen I-bound decorin could conceivably support TGF-β sequestration while also being deposited in the ECM. To elucidate how injected decorin regulates TGF-β activity, it would be informative in future studies to map the TGF-β binding sites in more detail and to express and use, in preclinical studies with fibrosis models, site-mutated variants of decorin that do not support TGF-β binding, in order to investigate to what extent modulation of TGF-β activity by decorin occurs through indirect mechanisms.

Stiffness and TGF-β drive the conversion of fibroblasts to αSMA-expressing fibroblasts that contract tissue.^60^^,^^61^ Such cells were significantly diminished in the forepaws of rh Decorin-treated RDEB mice. We also detected a reduction of damage-related, transient ECM proteins under the transcriptional control of TGF-β—tenascin-C and fibronectin,^62^^,^^63^—which also support ECM organization. Since our investigations unexpectedly revealed a reduction in the inter-molecular collagen crosslink DHLNL in RDEB mouse skin, it appears that fibrosis in the RDEB model is a consequence of cell-mediated contraction of the ECM, with additional support from transient ECM proteins. This makes TGF-β -modulating interventions to relieve fibrosis especially favorable for RDEB because reduction of αSMA-positive cells and transient ECM proteins, which are naturally turned over, could result in tissue relaxation and, consequently, improvement in the appearance of fibrotic lesions.

The approval of collagen VII gene therapies for RDEB has been a game changer for the management of the disease in countries where these options are available.^6^^,^^7^ However, there is variability in effectiveness and durability of response.^64^ There are likely many factors that influence these outcomes, but it is reasonable to suggest that the status of the dermal ECM and the general dermal microenvironment is a major factor—a more damaged and scarred dermis might have a lower ability to support collagen VII interactions needed for firm epidermal-to-dermal cohesion. Lowering tissue contractility and pro-fibrotic processes could create a dermis more supportive of proper integration of collagen VII-composed anchoring fibrils. Thus, pre- or co-treatment with agents that reduce the fibrotic response and promote dermal healing, such as decorin, could improve the response to and durability of curative therapies.

It should be pointed out that non-GAGylated decorin has been implicated in pathological processes. Mice unable to express GAGylated decorin were reported to present with skin thinning, fragility,^65^ and myofibrosis, which could be in alignment with studies suggesting that the lack of a GAG chain on decorin makes it less able to organize collagen fibrils.^26^ Loss of non-GAGylated decorin was found to increase in cardiac aging, and continuous systemic delivery of decorin with low levels of GAGylation resulted in cardiomyopathy,^66^ albeit the decorin used in these studies was not of clinical grade, and an arm of GAGylated decorin was not included. A truncated decorin form starting at leucine 51 and consequently lacking the GAG chain has been assigned pro-cancer effects.^67^ Nevertheless, to position such findings in the context of our work, some considerations have to be made. There is ample evidence from the literature that non-GAGylated decorin is therapeutically active, from treating fibroinflammation to cancer.^32^^,^^56^^,^^68^^,^^69^^,^^70^ In our study, non-GAGylated decorin effectively limited fibrosis in RDEB. It should also be noted that the first characterization of mice expressing only non-GAGylated decorin did not disclose tissue fragility and showed largely normal collagen fibrils and wound healing.^71^ Thus, the activity of non-GAGylated decorin in collagen fibril and matrix organization appears contextual. Our rationale was not to create a replacement for decorin in RDEB but to use therapeutic administration of decorin as an antifibrotic due to its ability to target many pathways associated with fibrosis. All these interactions are sustained by the protein core, and, in fact, these interactions might be even more potent due to the lack of the GAG chain.^15^^,^^16^

To conclude, here we show the activity of rh Decorin on specific RDEB-related signs, which is associated with accumulation of human decorin specifically at injured sites. We provide evidence that systemic administration of rh Decorin allows for potent targeting of TGF-β -activity in peripheral tissues. The short circulating half-life of rh Decorin and its natural retention in injured sites limit the risk of nonspecific tissue targeting and retention of rh Decorin. In conclusion, our data support the further testing of intravenous rh Decorin for the systemic treatment of RDEB, especially the fibrotic manifestations of the disease that are very debilitating for those affected by RDEB.

## Materials and methods

### Approval

The studies using collagen VII hypomorphic (RDEB-model) mice were approved by the regional ethics review board (Regierungspräsidium Freiburg) (approval no. G-22/059). Use of human archival donor-derived material was approved by the ethics committee of the University of Freiburg (Freiburg, Germany) (approval no. 425/14). Cells had been collected from the donors after the legal guardians had given informed written consent.

### Production of rh Decorin and analyses

Human decorin with a codon 34 mutation (TCT to GCT), resulting in a serine-to-alanine substitution, was expressed in CHO cells and purified by Catalent Pharma Solutions, Inc. as a paid service commissioned by Kamal Therapeutics. Analyses of the purified product and multiple batches were performed by Catalent Pharma Solutions, Inc. and Pace Analytical Life Sciences LLC, also as paid services by Kamal Therapeutics.

Native and denatured SDS-PAGE analyses with Coomassie staining were run under standard conditions. For western blotting, the mouse monoclonal anti-human decorin antibody MAB143 (R&D Systems) at a dilution of 1:50 was used.

Direct infusion ESI-MS experiments were performed with the following parameters: Sample preparation: 1 mg/mL hr Decorin buffer-exchanged into 20 mM ammonium acetate, pH 7.5, using 5,000 Da molecular weight cut-off filters. Syringe pump: 100 μL/min. ESI: spray voltage 3.5 kV; polarity positive; resolution 17,500; automatic gain control (AGC) target 1e6; max IT 250 ms; fragmentation 100 eV in-source collision-induced dissociation (CID); microscans 3; scan range 600–6,000 m/z; sheath flow gas 45; aux gas 15; capillary temp 350°C; S-lens RF 65; aux gas heater temp 150°C.

Capillary electrophoresis (CE)-SDS was performed on a Sciex PA800 system under reducing conditions. Samples were prepared according to standard methods using Sciex reagents. rhDecorin was diluted to 2 mg/mL into SDS sample buffer containing 2-mercaptoethanol. Samples were heated at 70°C for 10 min and then analyzed by CE on a Sciex PA800. The molecular weight of the peaks was assessed based on a molecular weight sizing standard and then calculated using the relative migration time to a 10 kDa internal standard.

Deglycosylation was performed with NEB PNGase F (P/N P0704S). rhDecorin (80 μg) was denatured with NEB glycoprotein denaturing buffer at 100°C for 10 min. Denatured samples were placed on ice and then briefly centrifuged. A reaction mixture was prepared by adding NEB glycobuffer 2, 10% NP-40, and water. Subsequently, 2,000 units of PNGase F (1:25 ratio) was added, and the mixture was incubated at 37°C for 90 min before analysis by CE or LC-MS.

Size-exclusion chromatography of challenged and forced-degraded rh Decorin was performed using a G3000SWXL column (Tosoh Bioscience) and 150 mM arginine in the mobile phase on a Shimadzu HPLC7 system.

LC-MS analysis was performed using an Agilent 1290 LC (Agilent Technologies) and a Q Exactive Plus MS (Thermo Fisher Scientific) using the following parameters: Acquisition dd-MSMS; capillary voltage 4.0 kV; capillary temperature 350°C; sheath gas 45; aux gas 15; S-Lens RF level 65; ionization mode positive; in-source CID 0 eV; default charge state 2; full MS microscans 1; resolution 70,000; AGC target 1e6; max IT 100 ms; number of scans 1; scan range 200–2000 m/z; spectrum data type profile; dd-MSMS/dd-SIM microscans 1; resolution 17,500; AGC target 1e5; max IT 200 ms; loop count 5; MSX count 1; TopN 5; isolation window 2.0 m/z; isolation offset 0.0 m/z; dd settings: minimum AGC threshold 1.00e3; charge exclusion 1,6–8; peptide match preferred; isotopes excluded; dynamic exclusion 10.0 s.

Analysis of acquired data was performed with Byos (Protein Metrics) software. The following conditions were used: enable lock mass calibration, false; apex time matching tolerance, 0.2; averaging correlation cut-off, 0.9; min feature mass, 500; max feature mass, 8000; min scan count, 3; min isotope count, 3; noise factor multiplier, 3; enable MS2 matching, 0; min peak width, 0.01 min; max precursor mass, 10,000; smoothing width (m/z), 0.01; precursor mass tolerance, 10 ppm; fragmentation type, QTOF/HCD; fragment mass tolerance, 40 ppm; cleavage site, RK; cleavage side, C-terminal; digestion specificity, fully specific; missed cleavages, 2; modifications: carbamidomethyl (+57.02164) @ C fixed; carbamidomethyl (+57.02164) @ N-terminal, rare (1); dicarbamidomethyl (+114.042927) @ N-terminal, rare (1); oxidation (+15.994915) @ N-terminal, common (1); deamidation (+0.984016) @ N-terminal, common (2), Gln→pyro-Glu (−17.026549) @ N-terminal Q, rare (1); Gln→pyro-Glu (−18.010565) @ protein N-terminal E, rare (1); ammonia-loss (−17.026549) @ Nterm C, N, rare (1); dehydrated (−18.010565) @ D, S, T, Y, rare (1); cation:Na (+21.981943) @ Cterm D, E, rare (1); dioxidation (+31.989829) @ M, W, rare (1); Lys-loss (−128.094963) @ protein Cterm K, rare (1); Hex (+162.052824) @ Nterm, K, rare (1); total common max, 2; total rare max, 1.

### Solid-phase binding assay

Solid-phase binding assays were performed as previously described.^47^ Briefly, 100 ng of collagen I from calf skin (IBFB GMBH), dissolved in 0.1% acetic acid, was immobilized per well on a NUNC Maxisorp 96-well plate at 37°C for 1 h. Between all subsequent steps, wells were washed three times with PBS to remove unbound ligand or antibody. The plate was then incubated with 0.5% BSA in PBS for 1 h to reduce nonspecific binding. Serial dilutions of rh Decorin, ranging from 1100 to 0.00011 nM, were incubated for 2 h. A primary antibody against decorin was applied at a 1:1000 dilution and incubated for 60 min. After incubation with a donkey anti-goat secondary antibody conjugated to horseradish peroxidase (1:1000 dilution) for 1 h, o-Phenylenediamine dihydrochloride (OPD) substrate (Sigma-Aldrich) was added, and absorbance was measured at 450 nm.

### Collagen fibrillogenesis assay

The assay was performed in 96-well microtiter plates. Per reaction, collagen I from calf skin was prepared at a final concentration of 1 mg/mL in 0.01% acetic acid, together with 1× PBS, with or without 160 nM rh Decorin. As a control, collagen I in 0.01% acetic acid diluted in water, without PBS or rh Decorin, was included. Reactions were mixed on ice, and 100 μL was added per well. Plates were incubated in a Tecan microtiter plate reader at 37°C, and absorbance at 405 nm was recorded every 2 min for 80 min.

### Treatment of mice

RDEB-model mice were injected with 100 μL PBS or rh Decorin at a concentration of 5 mg/mL via tail vein injections starting at 5 weeks of age. The mice received two injections per week, 3 days apart. The mice were weighed and observed daily and photographed weekly. In total, 6 mice received rh Decorin treatment and 5 received PBS treatment (3 females and 3 males and 2 females and 3 females, respectively). For histological and biochemical analyses, skin and paw samples from 4 PBS-treated and 6 decorin-treated mice were used. Samples from a PBS-treated mouse that had to be euthanized on day 27 were included in these analyses.

Quantification and scoring of mitten deformities were performed as previously described using Fiji software (NIH). The data were scored blinded.

### Histological and immunofluorescence analyses

Shaved back skin and forepaws were either embedded in optimal cutting temperature medium and snap-frozen for cryosectioning and western blotting or fixed in 10% formalin and processed for paraffin embedding. Per mouse, the forepaws were randomly assigned to either cryosectioning and western blotting or paraffin embedding. Paraffin sections were used for hematoxylin and eosin (H&E) and picrosirius staining. Staining with H&E was performed using standard protocols, and picrosirus red staining on 30 μm-thick sections was performed as previously described.^72^ The images of H&E-stained tissues were acquired under white light, and picrosirius red-stained tissue was imaged under polarizing light.^72^

For immunofluorescence staining, cryosections were used to stain for fibronectin, tenascin-C, decorin, CD11b, F4/80, and Ly6G. For αSMA and fibrillin-1, formalin-fixed, paraffin-embedded ^54^sections were used after antigen retrieval with citrate buffer and pepsin, respectively. The antibodies used were: rabbit anti-fibronectin (ab2413, Abcam), rat anti-mouse tenascin-C clone 578 from R&D Systems, anti-CD11b V450 clone M1/70 (BD Horizon, ref. 560455), anti-F4/80 PerCP/Cy5.5 clone BM8 (BioLegend, ref. 123128), anti-mouse Ly6G clone 1A8 (BioLegend, ref. 127618), goat anti-human decorin AF143 from R&D Systems, Cy3-conjugated mouse anti-human αSMA clone 1A4 (Thermo Fisher Scientific), and a previously described rabbit polyclonal fibrillin-1 antibody^54^ provided by Dr. Gerhard Sengle, University of Cologne.

Secondary antibodies used were: Alexa Fluor 594 goat anti-rat IgG (Invitrogen, ref. A11007) (1:1,000), Alexa Fluor 488 goat anti-rat IgG (Invitrogen, ref. A11006), and Alexa Fluor 488 donkey anti-goat IgG (Invitrogen, ref. A11055). Nuclei were counterstained with 4′,6-diamidino-2-phenylindole (DAPI).

The stains were quantified using Fiji software (NIH).

### Western blotting and ELISA

Tissue lysates were prepared by crushing snap-frozen skin or paws to a fine powder with a hammer and lysing the tissue in NP-40 lysis buffer (50 mM Tris-HCl, pH 8.0; 150 mM NaCl; 1% NP-40) with phosphatase and proteinase inhibitors. 30 mg of tissue was lyzed in 300 μL NP-40 buffer. After lysing under rotation for 40 min at 4°C, the lysates were cleared by centrifugation and used for western blotting or ELISA measurements of decorin levels.

To measure human decorin levels in mouse tissue and sera, a human-specific decorin ELISA assay (SEC127) from Cloud-Clone Corp. was used. For the measurement of sera and protein lysates, per well, 10 μL of sera or cleared tissue lysates were diluted in 90 μL PBS. The ELISA was performed and read according to the manufacturer’s instruction, and the concentration was calculated using a standard curve based on rh Decorin.

For western blotting, tissue lysates in NP-40 buffer were boiled in Laemmli loading buffer and loaded onto 10% SDS-PAGE gels. After separation, proteins were transferred onto nitrocellulose membranes, which were blocked and probed with rabbit anti-pSMAD-2 (Ser 465/467) (138D4), rabbit anti-pSMAD-3 (S423/425) (ab52903), rabbit anti-fibronectin (ab2413, Abcam), mouse anti-human αSMA clone 1A4, rat anti-mouse tenascin-C clone 578, goat anti-human decorin AF143, rabbit anti-fibrillin-1,^54^ and rabbit anti-β-tubulin (ab6046). For the blot shown in Figure 1A, recombinant human decorin was purchased from Hycultec (HY-P7885). Western blots were densitometrically quantified using Fiji software.

### Collagen crosslink analysis

The analysis of collagen crosslinks was performed as previously described using an amino acid analyzer (Biochrome 20).^73^ The nomenclature used in the text refers to the reduced variants of difunctional intermediate cross-links (DHLNL, HLNL). For the analysis, 5 mm punch biopsies of shaved back skin from 6 female wild-type and 6 female RDEB-model mice were used; the mice were between 9 and 11 weeks old at the time of collection. Back skin was used since it was not possible to reliably obtain the amount of material needed for the analyses from forepaw skin. Although back skin is spared from the development of macroscopic fibrosis, at 9–11 weeks of age, back skin of RDEB-model mice display molecular signs of progressed fibrosis^33^ and female mice were used to avoid variations from biological sex.

### Analysis using RDEB-donor fibroblasts

Human dermal fibroblasts isolated from one juvenile donor with generalized severe RDEB due to complete collagen VII deficiency was used^48^; approved by the ethics committee of the University of Freiburg (Freiburg, Germany) (approval no. 425/14). The cells were seeded to confluence either on coverslips or in 6-well plates wells and grown in DMEM with 10% fetal calf serum (FCS). For the assessment of short-term impact of rh Decorin on basal TGF-β activity, cells were treated 10 h after seeding with 4 μg/mL rh Decorin or an equal volume of PBS. After 1 h, the cells were harvested and processed for western blotting as described below. For the 4-day culture experiment, the day after seeding and for 3 consecutive days, the fibroblasts were treated with 4 μg/mL rh Decorin or an equal volume of PBS. After 4 days in culture, the cells were either processed for western blotting by incubation with NP-40 lysis buffer followed by collection with a cell scraper for preparation of cell and matrix lysates, or, for analysis by immunofluorescence staining, fibroblasts on coverslips were fixed in 4% paraformaldehyde. Fibrillin-1 abundance or fibrillin-1 deposition was analyzed by western blotting or immunofluorescence staining using standard conditions as described above and a rabbit polyclonal fibrillin-1 antibody.^54^ *FBN1* gene expression was analyzed by qPCR using the previous described protocol and primers.^48^

Free-floating collagen-gel contraction assay was performed as previously described.^54^ After release of the gels, 4 μg/mL rh Decorin or an equal volume of PBS was added daily, and photos were captured after 3 days (72 h). The collagen gel area was quantified using ImageJ.

### Statistical analyses

Data were analyzed using Graphpad Prism 10. The data were first tested for normality and equal variance using Shapiro-Wilk and F tests. The following tests were used: ANOVA mixed-effects analysis; for Figure 2B, ANOVA mixed-effects analysis with Šídák’s multiple comparisons test; Figure 2C, log-rank (Mantel-Cox) test; Figure 3A, Mann-Whitney test; Figure 3B, Kruskal-Wallis with uncorrected Dunn’s test; Figure 3C, Mann-Whitney; Figures 3E–3G, Student’s unpaired *t* test; Figure 4B, Mann-Whitney; Figure 4C, Kruskal-Wallis test with Dunn’s multiple comparisons test; Figure 5C, Student’s unpaired *t* test; Figures 6A–6C and 6E, Student’s unpaired *t* test; Figure 6G, Student’s paired *t* test; Figure 6H, simple linear regression; Figure S3, non-linear regression (one-site specific binding); and Figures S1–S7, Student’s unpaired *t* test. The data are presented as means with standard errors of the mean.

## Data and code availability

All data are presented in this paper and its supplemental information.

## Acknowledgments

The work was supported by research grants from Cure EB and the Office of the Assistant Secretary of Defense for Health Affairs, through the Peer Reviewed Medical Research Program under award no. W81XWH1910834. Both grants were awarded to Kamal Therapeutics. The Opinions, interpretations, conclusions, and recommendations are those of the authors and are not necessarily endorsed by the 10.13039/100000005Department of Defense. The 10.13039/100014055U.S. Army Medical Research Acquisition Activity, 839 Chandler Street, Fort Detrick, MD 21702-5014, is the awarding and administering acquisition office. In conducting research using animals, the investigator(s) adhered to the laws of the United States and regulations of the Department of Agriculture. In the conduct of research utilizing recombinant DNA, the investigator adhered to 10.13039/100000002NIH Guidelines for research involving recombinant DNA molecules. In the conduct of research involving hazardous organisms or toxins, the investigator adhered to the CDC-NIH Guide for Biosafety in Microbiological and Biomedical Laboratories.

In addition, the work was supported by research grants from the 10.13039/501100001659Deutsche Forschungsgemeinschaft (DFG, 10.13039/501100001659German Research Foundation) project nos. 397484323 (TRR259/B09), 468236352, and 384170921 (FOR2722/C01) to G.S. and NY90/5-1, NY90/6-1, and CRC1160 project B03 to A.N. The funders had no role in study design, data collection and analysis, or decision to publish the findings.

## Author contributions

Conceptualization, M.P.d.S., H.L., and A.N.; experimentation, C.G., B.H., B.B., S.F., A.B., G.B., I.J.C., J.B., and A.N.; data interpretation, C.G., B.H., G.S., C.P., B.B., S.F., A.B., G.B., I.J.C., J.B., and A.N.; providing key materials, G.S., C.P., M.P.d.S. and H.L.; funding acquisition, M.P.d.S. and G.S.; supervision, C.P., M.P.d.S., H.L., and A.N.; manuscript writing, A.N.; manuscript editing, C.G., B.H., G.S., C.P., J.B., M.P.d.S., H.L., and A.N.

## Declaration of interests

B.B., S.F., and A.B. are employees of Pace Laboratories with a yearly financial benefit exceeding $10,000. G.B. and I.J.C. are employees of Catalent Pharma Solutions Inc. with a yearly financial benefit exceeding $10,000. C.P., M.P.d.S., and H.L. are consultants to Kamal Therapeutics with a yearly financial benefit exceeding $10,000. Kamal Therapeutics funded the work at Catalent and Pace Laboratories, along with the DoD.

## Supplemental information

Document S1. Figures S1–S7

Table S1. List of post-translational modifications in two batches of rh Decorin—G001 and 09018 N- and O-linked glycans are shown in Table S2.

Table S2. List of all identified N- and O-linked glycans in two batches of rh Decorin—G001 and 09018, including the relative abundance of each modification for each peptide

Document S2. Article plus supplemental information
