# Supplementary material for: Systemic clinical-grade recombinant decorin reduces disease burden and fibrosis in advanced recessive dystrophic epidermolysis bullosa
Source: Mol Ther Adv. 2026 Apr 15;34(2):201737. doi: 10.1016/j.omta.2026.201737 (PMC13175769; doi:10.1016/j.omta.2026.201737)
Supplement: Document S1. Figures S1–S7 [file mmc1.pdf]

OMTA, Volume *34*

Supplemental information

Systemic clinical-grade recombinant decorin reduces disease burden and fibrosis in advanced recessive dystrophic epidermolysis bullosa

Christine Gretzmeier, Bing Hang, Gerhard Sengle, Christopher Phillips, Ben Buer, Sarah Fletcher, Arseniy Belov, Gregory Bleck, Ian J. Collins, Jürgen Brinckmann, Mark P. de Souza, Hal Landy, and Alexander Nyström

Supplemental information – Gretzmeier, Hang et al., 2026

Table S1 List of post-translational modifications in two batches of rh Decorin – G001 and 09018. N- and O-linked glycans are shown in Table S2.

Table S2 List of all identified N- and O-linked glycans in two batches of rh Decorin – G001 and 09018, including the relative abundance of each modification for each peptide.

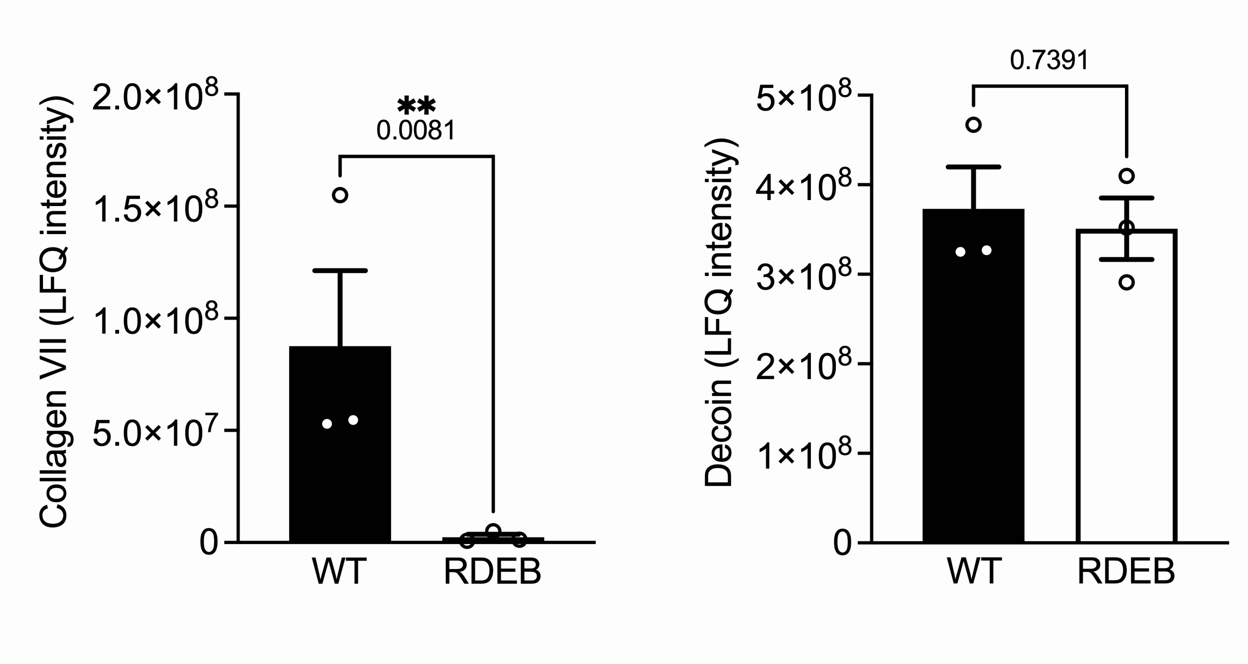

Figure S1. Decorin is not reduced in back skin from adult RDEB model mice. Data from mass spectroscopy-based proteomics of back skin from 10-week-old wild-type (WT) or collagen VII hypomorphic (RDEB) mice (Bernasconi et al., EMBO Mol Med 2021), analyzed for collagen VII and decorin abundance. Statistical analysis was performed using unpaired Student's t-test. Data are presented as mean ± SEM. P values as indicated. ** $P < 0.01$.

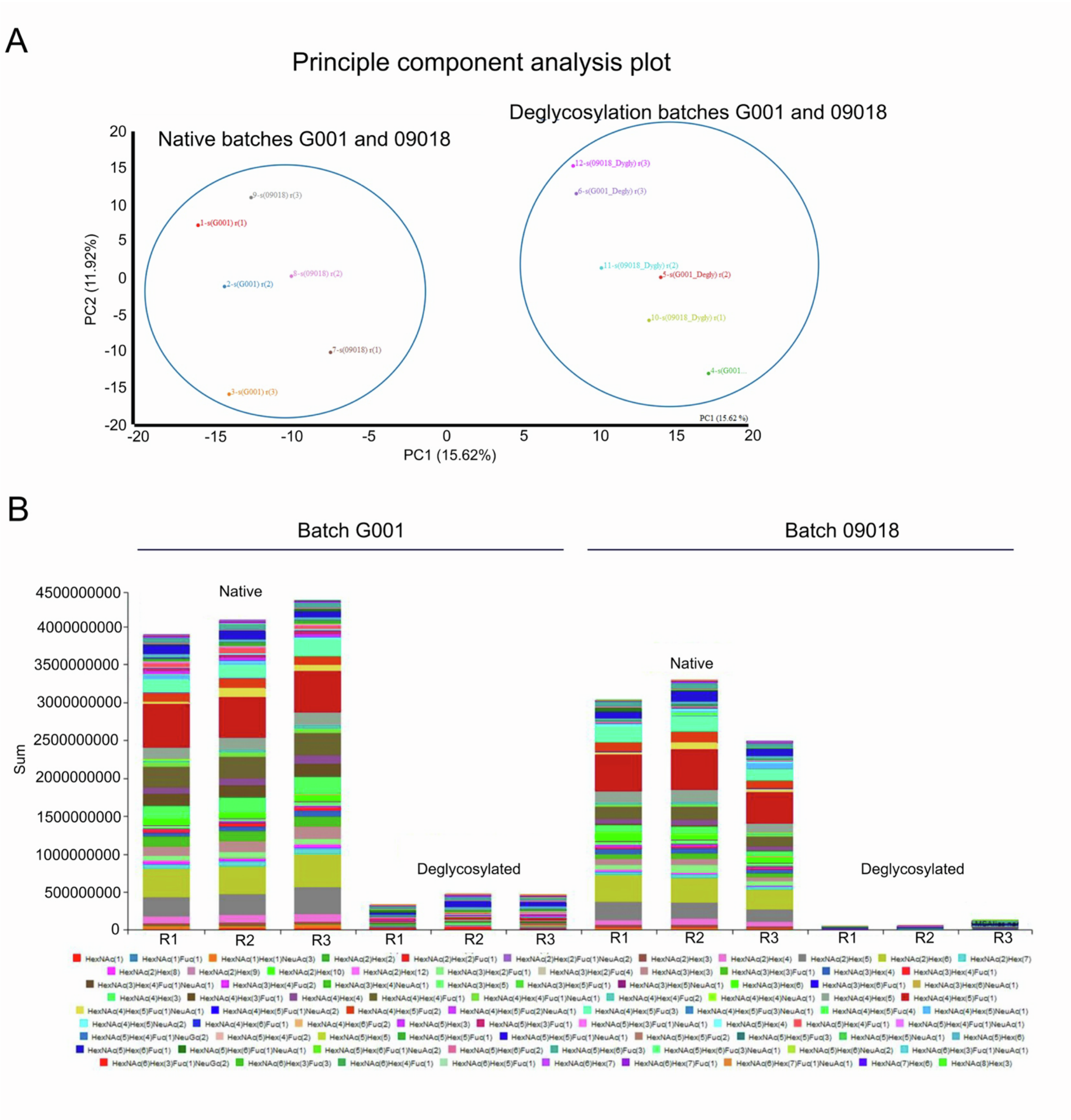

Figure S2. Low batch variability of rh Decorin. A-B, Two batches of rh Decorin, prior or after deglycosylation under denaturing conditions, analyzed by mass spectrometry. A, Principal component analyses. B, Plot of detected glycosylations.

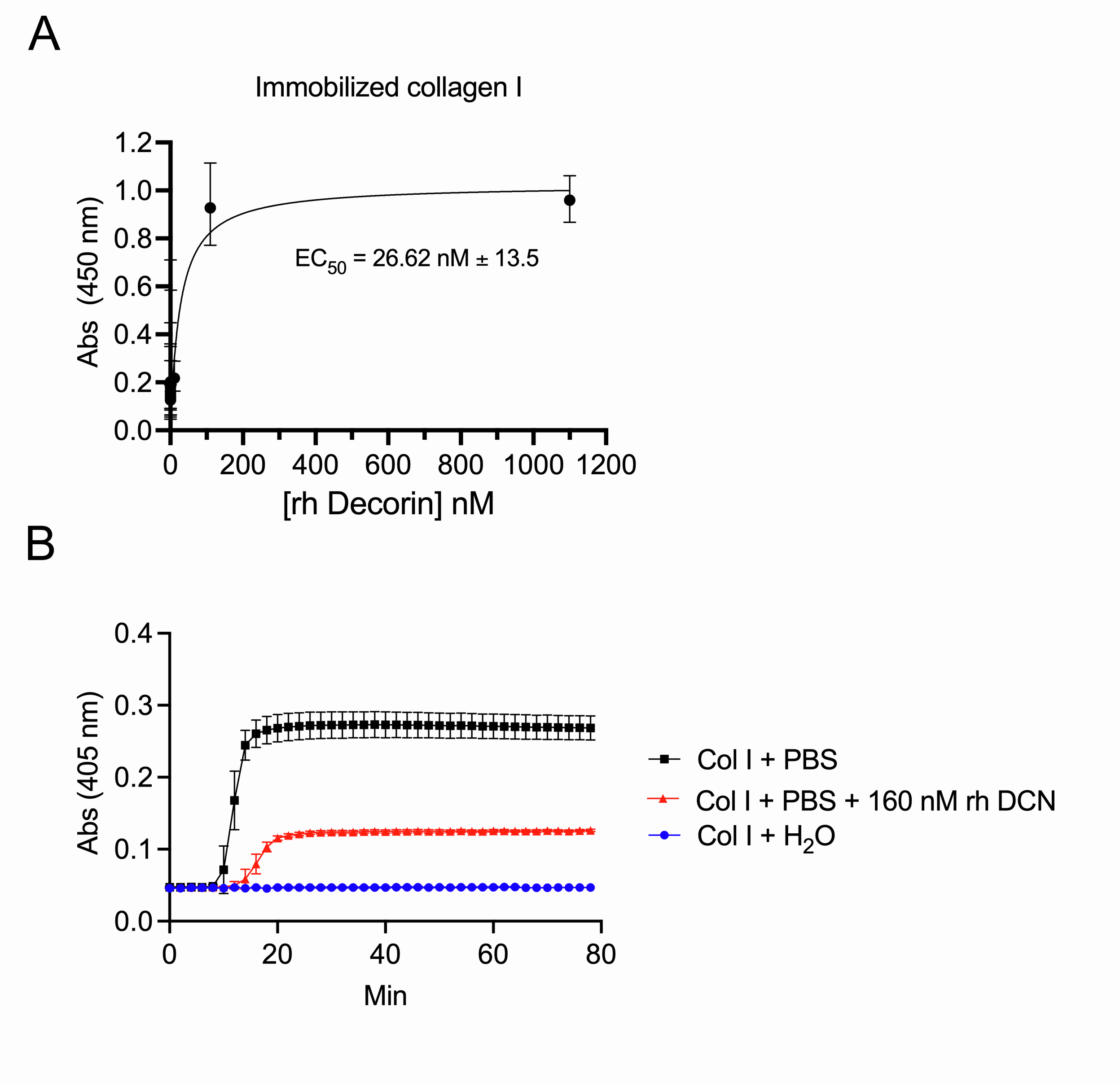

Figure S3. rh Decorin binds collagen I and delays collagen fibrillogenesis in vitro. A, Solid-phase binding assay of rh Decorin to immobilized calf skin collagen I. EC50 value ± SEM is indicated. B, Collagen fibrillogenesis (turbidity) assay. Fibrillogenesis was monitored in vitro under the indicated conditions by measuring turbidity at 405 nm over time following incubation at 37 °C.

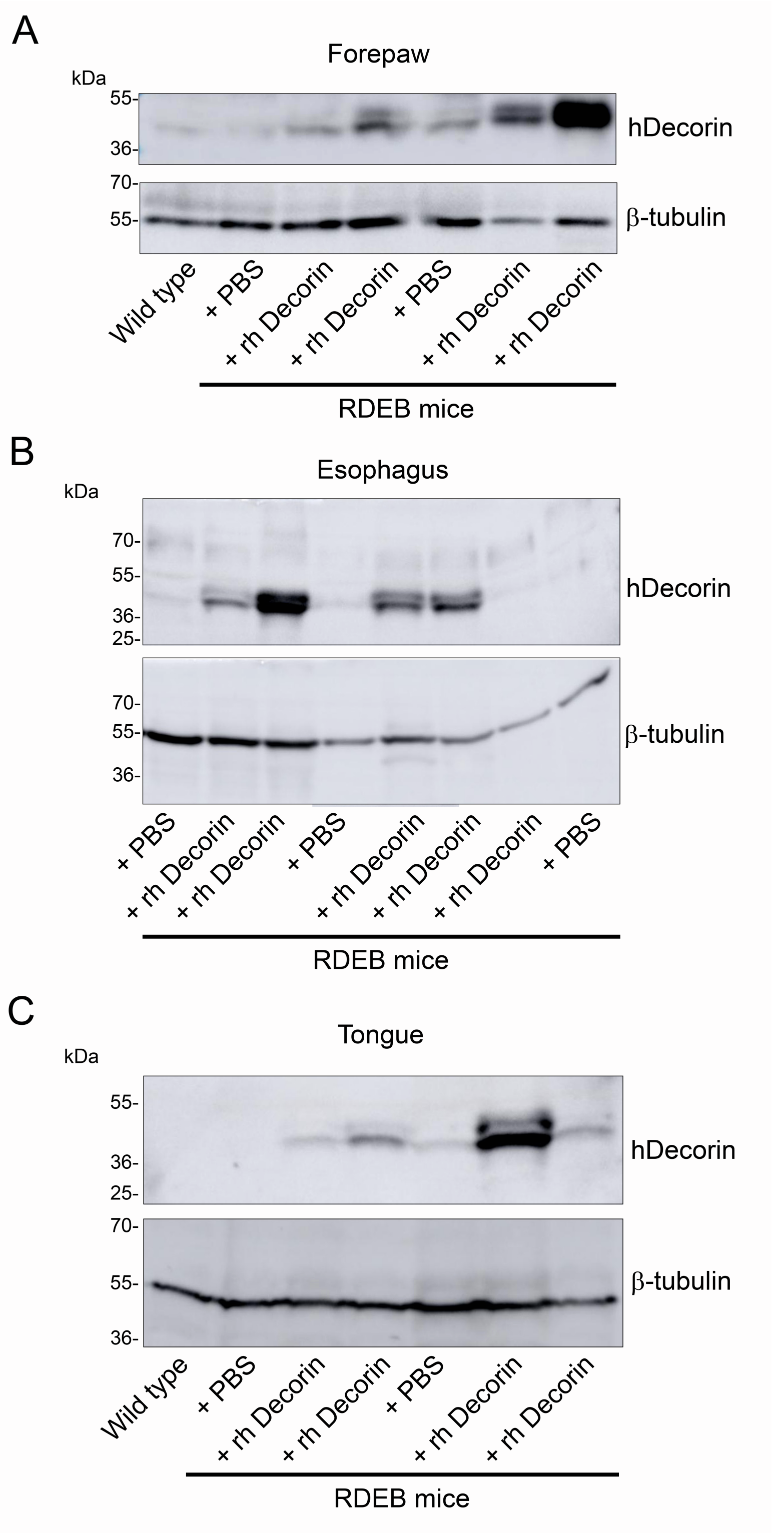

Figure S4. rh Decorin accumulates in damaged tissue. A-C Western blotting of tissue lysates from forepaws (A), esophagus (B) and tongue (C) from age-matched wild-type or RDEB mice after treatment with PBS or rh Decorin for four weeks. The blots were probed with an antibody with preferential reactivity toward human decorin and and β-tubulin as a loading control. Two lanes for blot A are also shown in Figure 6F.

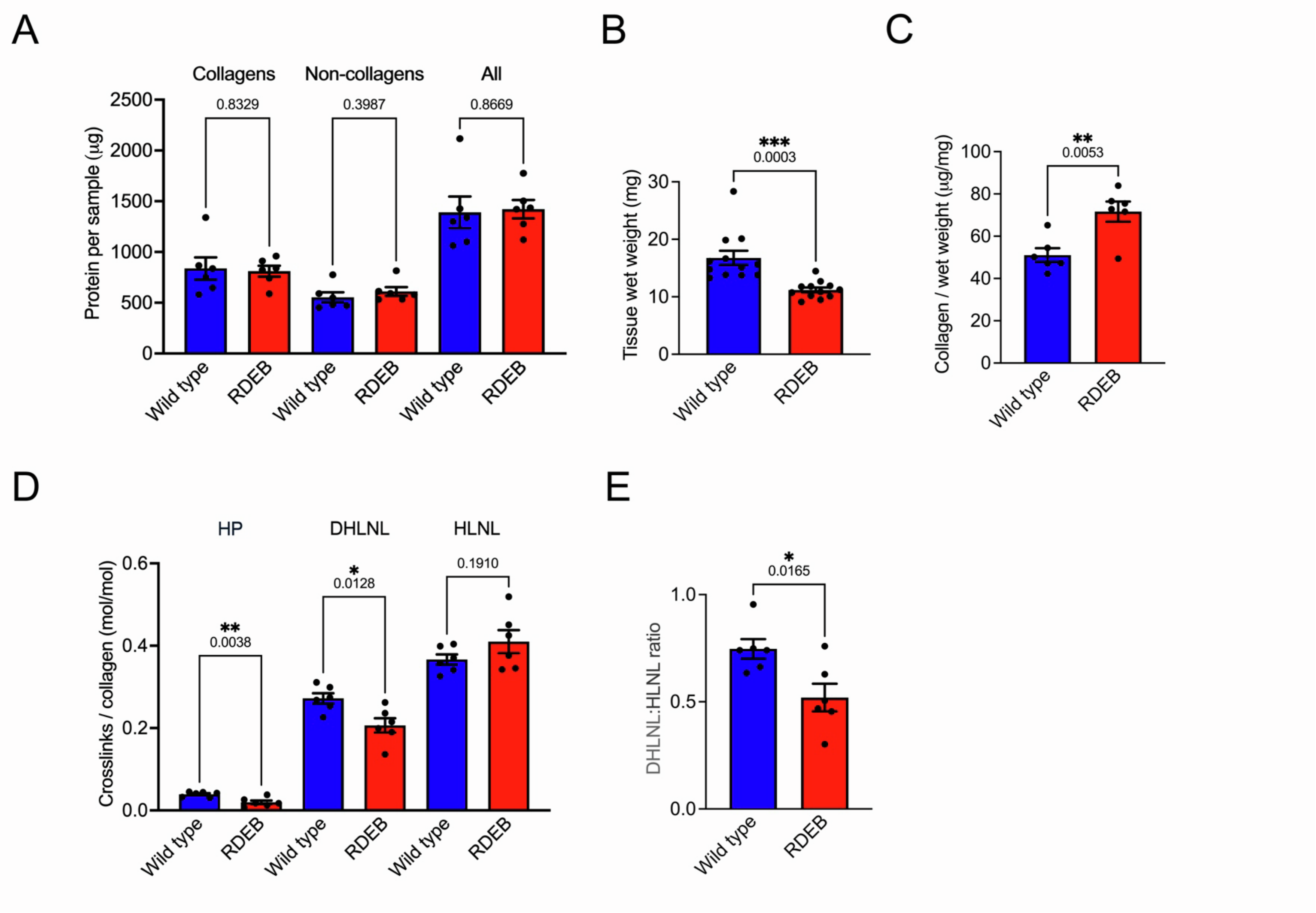

Figure S5. Reduction of water content and collagen crosslinks in skin from RDEB mice with advanced disease. A, Quantity of collagens, non-collagen proteins and total protein content in back skin of 9-11-week-old female wild-type and RDEB mice. B, Total wet weight of 5 mm punch biopsies of back skin from mice as in A. C, Collagen content vs. wet-weights in skin biopsies from wild-type mice and RDEB mice as in A. D, Collagen crosslinks – hydroxylysylpyridinoline (HP) and dehydrodihydroxylysinonorleucine (DHLNL) and hydroxylysinonorleucine (HLNL) measured in samples as in A. E, Plot of DHLNL:HLNL ratio. Statistical analysis was performed using unpaired Student's t-test. Data are presented as mean ± SEM. P values as indicated. *$P < 0.05$, ** $P < 0.01$,*** $P < 0.001$.

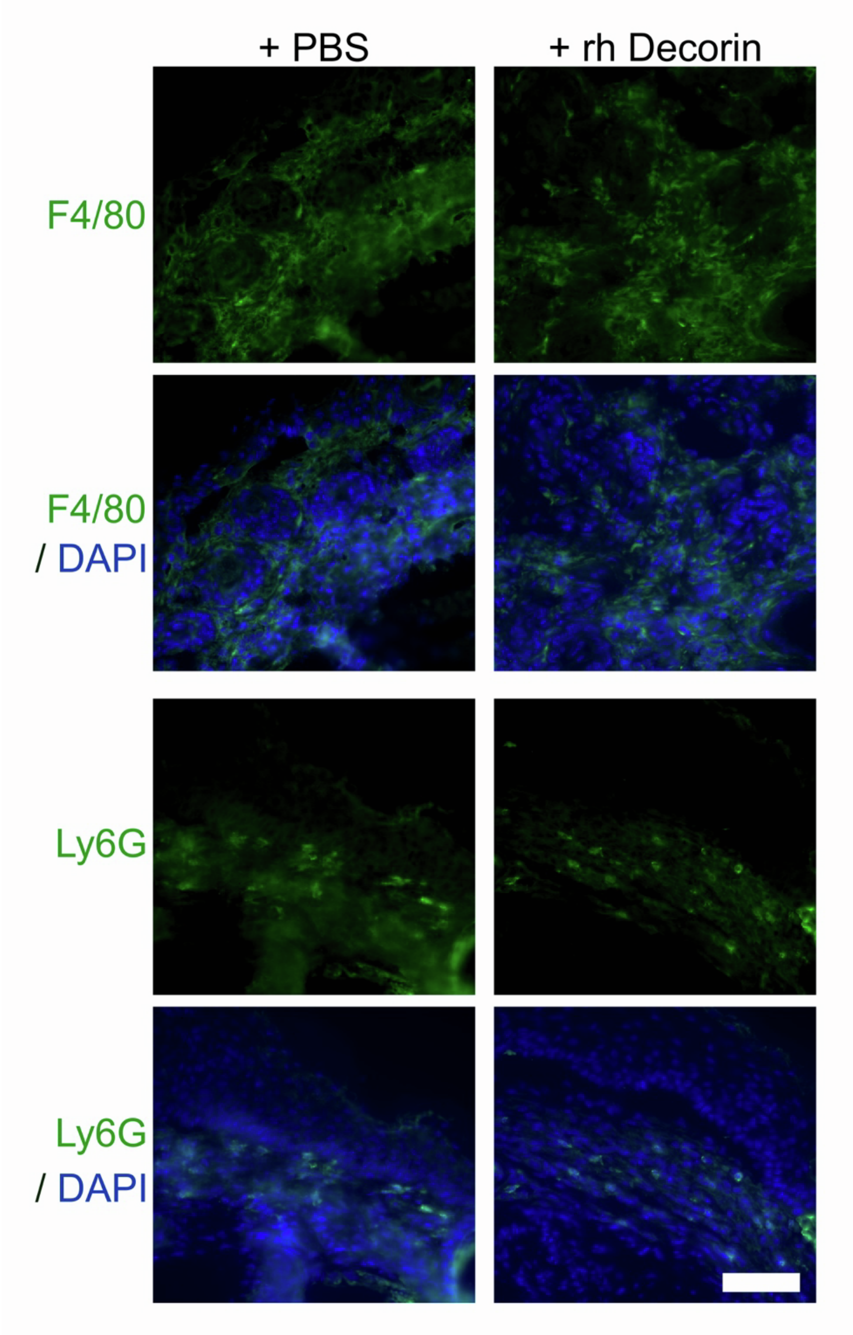

Figure S6. Limited impact on macrophage and neutrophil infiltration by rh Decorin treatment. Sections of forepaws from RDEB mice treated with PBS or rh Decorin for four weeks stained for F4/80 (green, macrophage marker) and Ly6G (green, neutrophil marker). Nuclei stained with DAPI. Scale bar = 50 μm.

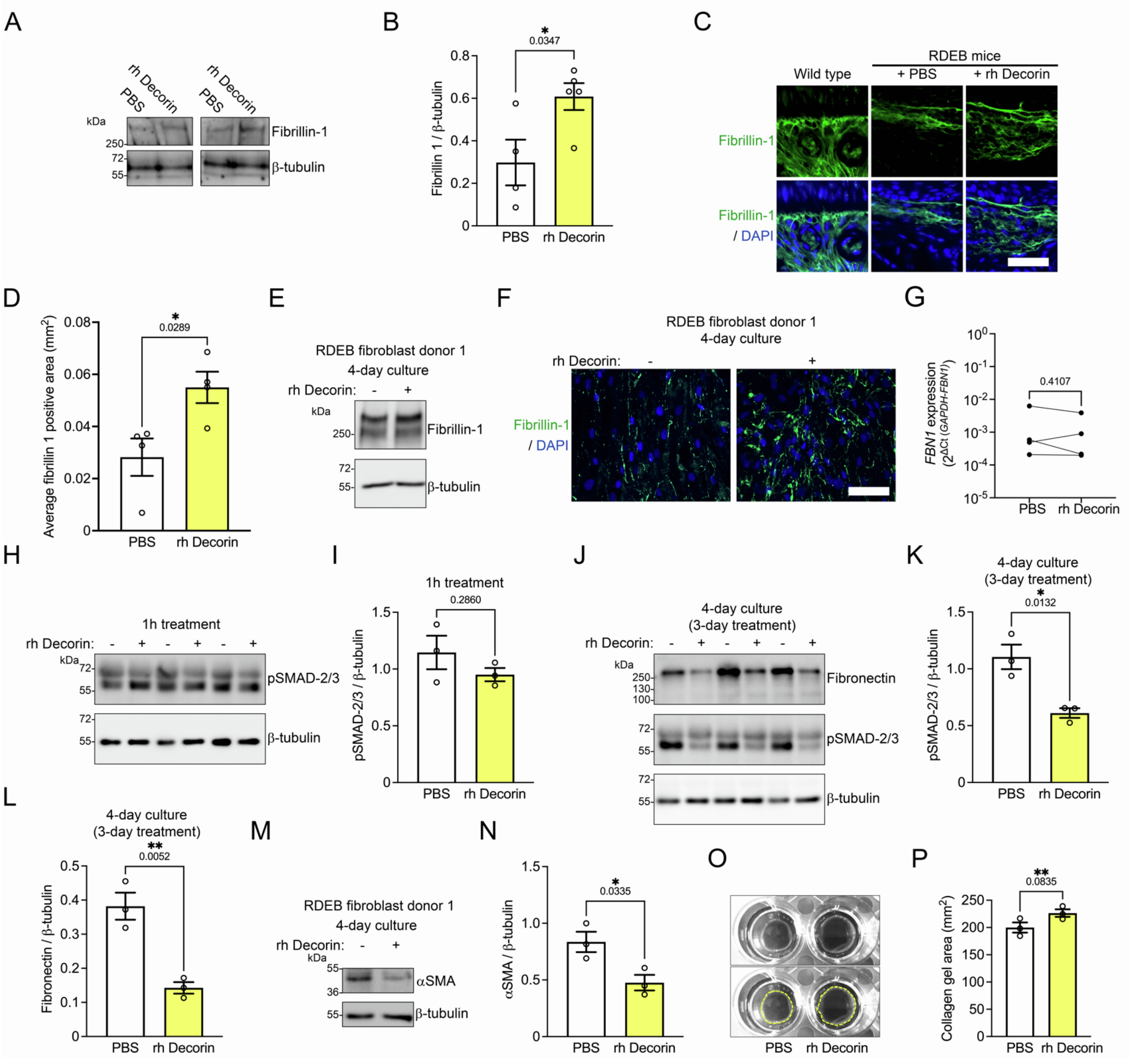

Figure S7. rh Decorin increases fibrillin-1 abundance and the appearance of dermal fibrillin-1 microfibrils in RDEB. A, Western blot for fibrillin-1 and β-tubulin of whole forepaw protein lysates from RDEB-model mice treated with biweekly PBS or rh Decorin injections for four weeks. B, Quantification of fibrillin-1 abundance normalized to β-tubulin in blots as in A. C, Staining of fibrillin-1 (green) and DAPI (blue) in forepaw sections from age-matched wild-type and RDEB-model mice treated as in A. D, Quantification of stained fibrillin-1 area in sections as in C after application of an equal threshold. E, Western blot for fibrillin-1 and β-tubulin of lysates from

one human RDEB-donor derived dermal fibroblasts daily treated with vehicle (PBS) or 4 μg/ml rh Decorin and cultured for four days. F, Staining of fibrillin-1 (green) deposition in fibroblast cultures as described in E. Nuclei were visualized with DAPI (blue). G, RT-qPCR of fibrillin-1 gene expression (*FBN1*) normalized to *GAPDH* in RDEB-donor derived fibroblasts treated as in E. Western blot of RDEB-donor fibroblasts from three different donors treated with 4 μg/ml rh Decorin for 1h. The cells were seeded the day before seeding. Blots probed for pSMAD-2/3 and β--tubulin. I, Densitometric quantification of blot as in H. J, RDEB fibroblasts as in H, treated with 4 μg/ml rh Decorin for three consecutive days after seeding and harvested for analyses one day after the last treatment. Blots probed for pSMAD-2/3, fibronectin, and β-tubulin. K and L, Densitometric quantification of blots as in J. M, Blots for αSMA of RDEB fibroblasts treated as in J. N, Densitometric quantification of blots as in M. O, Collagen gel contraction assay, RDEB fibroblast-populted free floating-collagen gels were treated with 4 μg/ml rh Decorin or an equal volume of PBS and followed for 72 h. Photos after 72 h shown, top and bottom row show the same photo but with the gel edges indicated with a yellow dotted line in the bottom row. Treatments are indicated. P. Quantification of gel contraction in experiments as in O. N = three donors in technical duplicates. Individual values are shown. Statistical analysis was performed using unpaired Student's t-test. Data are presented as mean ± SEM. P values as indicated. $^{*}P < 0.05$, Scale bars = 50 μm.
